# The Effects of Co-Exposure to Antifoulants and Microplastics on the Survival, Oxidative Status, and Cholinergic System of a Marine Mysid

**DOI:** 10.3390/toxics12090651

**Published:** 2024-09-05

**Authors:** Somyeong Lee, Md. Niamul Haque, Do-Hee Lee, Jae-Sung Rhee

**Affiliations:** 1 Department of Marine Science, College of Natural Sciences, Incheon National University, Incheon 22012, Republic of Korea; 2 Research Institute of Basic Sciences, Incheon National University, Incheon 22012, Republic of Korea; 3 Yellow Sea Research Institute, Incheon 22012, Republic of Korea

**Keywords:** marine mysid, metal-based antifoulant, microplastic, oxidative stress, antioxidant response

## Abstract

Antifoulants such as copper pyrithione (CuPT) and zinc pyrithione (ZnPT) are widespread and hazardous pollutants in aquatic environments. The presence of microplastics (MPs) introduces significant uncertainty regarding the toxicity of CuPT and ZnPT, as their effects can be influenced by MPs. There is a limited understanding of the toxic potential of CuPT and ZnPT when they coexist with MPs. Here, the marine mysid *Neomysis awatchensis* was treated using no observed effect concentration (NOEC) values of CuPT and ZnPT premixed with MPs (1 µm; 1–100 particles mL^−1^). The presence of MPs increased the toxicity of the antifoulants in juvenile and adult mysids over 96 h. The additive effect of the MPs varied by chemical; feeding was only reduced by CuPT with MPs, whereas no fluctuation in feeding was observed in response to ZnPT with MPs. Co-exposure to antifoulants and MPs increased malonaldehyde levels, but the response of antioxidant components varied by chemical. In mysids co-exposed to CuPT and MPs, the activity levels of catalase and superoxide dismutase were decreased, whereas their enzymatic activity levels were elevated by co-exposure to ZnPT and MPs. Similarly, depletion of glutathione (GSH) was observed in mysids co-exposed to CuPT and MPs, with significant reductions in GSH reductase (GR) and peroxidase (GPx). However, the GSH level was increased by co-exposure to ZnPT and MPs, with elevations in GR and GPx activity levels. Significant inhibition of acetylcholinesterase activity was only observed in response to CuPT and MPs. These results suggest that MPs can increase toxicity via additive and/or synergistic effects through oxidative imbalance, but these effects of MPs can vary with different chemicals.

## 1. Introduction

Biofouling refers to the colonization of diverse aquatic animals such as bacteria, algae, barnacles, and mussels on submerged surfaces like ship hulls, artificial structures in mariculture, and floating platforms. This phenomenon leads to substantial economic losses and environmental impacts [1]. Antifouling paints and coatings incorporating copper pyrithione (CuPT) and zinc pyrithione (ZnPT) have been extensively used to protect the natural and artificial surfaces of ships and aquatic structures against fouling organisms (e.g., bacteria, microalgae, invertebrates), acting as potential replacements for organotins, which were banned due to their significant toxicity to non-target organisms [2,3]. Due to the higher efficiency of metal-based antifouling agents, resulting from low water solubility, broad bactericidal, algicidal, fungicidal, and antimicrobial actions, and favorable environmental chemistry for numerous applications, the use of CuPT and ZnPT has expanded dramatically worldwide [4]. While environmental concentrations of ZnPT have been predicted to reach up to 0.4 μg L^−1^ in marine environments [5], there is limited information available on the actual levels of CuPT and ZnPT detected in waterbodies.

Due to the extensive production, sale, use, and emerging applications of CuPT and ZnPT, research has been conducted to estimate their harmful potential towards aquatic organisms. These studies have reported increased mortality, reduced growth or hatching rates, developmental deformities, and physiological and histological alterations in response to CuPT or ZnPT [6,7,8,9,10,11,12,13,14]. In particular, the hazardous effects of CuPT and ZnPT were reported in a marine mysid through the analysis of acute toxicity assay, survival and growth measurement with analysis of intermolt duration, feeding activity, and the number of newborn juveniles across three generations, along with biochemical alterations in glutathione S-transferase (GST) and the cholinergic indicator acetylcholinesterase (AChE) [15]. Since CuPT and ZnPT can be consistently released from antifouling paints into marine environments, numerous substances floating or dissolved in waterbodies can be directly or indirectly mixed with these antifoulants. However, no research has been conducted on their additive and/or synergistic effects with such substances on marine animals.

Among the many forms of anthropogenic pollution, plastic contamination is one of the emerging and severe global issues affecting aquatic ecosystems and threatening food safety [16,17]. In aquatic systems, various plastics can gradually fragment into microplastics (MPs) and/or nanoplastics (NPs) due to exposure to natural environmental and physical factors such as waves, heat, ultraviolet radiation, and biological degradation [18]. As numerous compounds are released into the aquatic environment directly or through wastewater discharge, the role of MPs/NPs as potential vectors of various co-existing substances into organisms, and their additive or synergistic effects, are of great concern beyond their sole effects [19,20,21]. Basically, plastics have a strong adsorption capacity for organic and inorganic compounds due to their significant binding capacity, hydrophobicity, and large surface area [22,23]. To the best of our knowledge, studies on the additive or synergistic effects of MPs on the toxicities of the two antifoulants in question have not been conducted yet, and these must be clearly understood.

In this study, to investigate whether MPs have additional or combined effects on the toxicities of CuPT and ZnPT, a marine mysid was employed as an experimental model, representing a small crustacean that is a non-target for antifoulants. Mysids are well-suited for toxicity analysis for several reasons: they have a widespread geographic distribution from pole to pole with significant abundance, are easy to handle and transport, possess a relatively short life cycle lasting between days and weeks, exhibit observable physiology, and show sensitivity to pollutants. These characteristics make mysids a model organism in ecotoxicological investigations [24,25]. In addition, mysids are essential food items at trophic levels that can influence the flow of nutrients and pollutants, thus playing a vital role in the transfer of energy in food webs. Their shrimp-like physiological and phenotypical aspects can represent environmental impacts on krill and other malacostracans.

In general, understanding how organisms respond to toxicity often involves analyzing biochemical endpoints in addition to observing mortality [26,27,28]. The measurement of oxidative stress and antioxidant response can deepen our understanding of biochemical responses in aquatic animals exposed to toxicants; this involves the analysis of crucial biomarkers such as malondialdehyde (MDA), glutathione (GSH), glutathione peroxidase (GPx), glutathione reductase (GR), superoxide dismutase (SOD), and catalase (CAT) [29,30,31]. AChE activity is commonly monitored as an indicator of toxic effects on behavior due to its crucial role in the cholinergic system. AChE terminates synaptic transmission by catalyzing the hydrolysis of the neurotransmitter acetylcholine (ACh) into choline and acetate [32].

To understand the potential additive or synergistic effects of MPs on CuPT and ZnPT, individuals of the marine mysid species *Neomysis awatchensis* were co-exposed to NOEC levels of CuPT and ZnPT along with MPs at concentrations of 1, 10, and 100 particles mL^−1^. Physiological alterations were assessed by analyzing feeding rate, with AChE activity as a supportive biochemical response. The biochemical responses of antioxidant defense components were analyzed to predict whether MPs can enhance the toxic effects of CuPT and ZnPT.

## 2. Materials and Methods

### 2.1. Animal Maintenance

The marine mysid *Neomysis awatschensis* population used in this study was continuously cultivated using artificial seawater (ASW) (TetraMarine Salt Pro; Tetra, Cincinnati, OH, USA) in an automated aquaculture system at Incheon National University (Incheon, Republic of Korea). Water parameters, including pH, dissolved oxygen (DO), salinity, and conductivity, were regularly assessed using a Orion^TM^ Star A Potable Meter (Thermo Fisher Scientific Inc., Waltham, MA, USA) equipped with pH/DO/conductivity electrodes. The environmental conditions were consistently maintained at a temperature of 20 °C, salinity of 30 practical salinity units (psu), a pH ranging from 7.9 to 8.2, DO concentrations between 6.8 and 7.3 mg L^−1^, and a light:dark photoperiod of 16:8 h. The mysids were fed with 70–100 *Artemia* nauplii (SERA *Artemia*, Salt Lake, UT, USA) per mysid twice daily.

### 2.2. Exposure

CuPT [C_10_H_10_CuN_2_O_2_S_2_, 1–hydroxy–2(1h)–pyridinethione, copper] and ZnPT (C_10_H_8_N_2_O_2_S_2_Zn, 1–hydroxypyridine–2–thione zinc salt) were obtained from ACROS Organics™ (Fairlawn, NJ, USA) and Merck KGaA (Darmstadt, Germany), respectively. Stock solutions of CuPT and ZnPT were created by dissolving each compound in dimethyl sulfoxide (DMSO; Sigma–Aldrich Co, St. Louis, MO, USA) and were stored in dark environments until use to minimize potential photodissociation. Working solutions were set by diluting each stock solution in 0.22 μm filtered ASW.

The toxicity values for both antifoulants were determined in our previous study [15]. The NOEC value in response to CuPT exposure was 1.24 ng L^−1^ for juveniles and 34 ng L^−1^ for adult mysids. Regarding ZnPT, the NOEC value was 24 ng L^−1^ for juveniles and 89 ng L^−1^ for adult mysids.

Non-functionalized polystyrene MPs with a mean diameter of 1 μm were purchased as aqueous suspensions from Sigma-Aldrich, Inc. (St. Louis, MO, USA). According to the manufacturer, the calibrated particle diameter ranged from 1.00 to 1.20 μm. The suspensions were slightly mixed with a vortex before sample dilution and exposure, and no additional surfactant was employed during the dilution procedure. For exposure, working solutions at specified nominal concentrations were created by dilution of the stock solution in 30 psu ASW.

The exposure criteria were based on our previous study [33]. A significant impact of MP concentration on survival rate for 1 and 10 μm of polystyrene MPs was observed at exposure concentrations above 5 × 10^4^ particles mL^−1^.

The overall methods followed for exposure to the two antifoulants and the MPs were those used in our previous studies [15,33]. Juveniles (<24 h after hatching) and adults were subjected to various concentrations of CuPT (0–2 μg L^−1^) or ZnPT (0–200 μg L^−1^) in the absence or presence (1, 10, or 100 particles mL^−1^) of 1 μm MPs, and a static 96 h acute toxicity test was conducted. In total, 30 mysids were utilized and divided into three sub-groups per concentration for CuPT or ZnPT treatment. The three groups were used in triplicate for statistical analysis. They were individually housed in 500 mL test vessels (Duran, Wertheim, Germany), each containing 300 mL of artificial seawater (ASW) for control, CuPT, or ZnPT treatments. Dead mysids found during the acute toxicity assay, identified by immobilization and lack of response from their antennae and swimming legs upon physical stimulation or artificial current, were immediately discarded from the assay. No mortality was observed in the control group throughout the acute toxicity assay.

### 2.3. Feeding Rate

The mysids were exposed to NOEC values of CuPT or ZnPT, in the absence or presence (1, 10, or 100 particles mL^−1^) of 1 μm MPs, for 96 h. The overall treatment conditions were consistent with those of the acute toxicity test. Feeding rate measurements were conducted with 12 juvenile mysids per treatment using 500 mL test containers (Duran), each containing 300 mL ASW (n = 1 mysid per container). *Artemia* nauplii, with an average size of 0.39 ± 0.07 mm, were used as prey. The number of *Artemia* nauplii consumed by juvenile mysids was measured for 1 h.

### 2.4. Analysis of Oxidative Stress and Antioxidant Parameters

To measure the fluctuations of biochemical parameters, a total of 210 mysids were used for each parameter. They were randomly separated into seven groups (30 mysids per time point) to prepare samples for seven time course collections (0, 12, 24, 48, 72, and 96 h, with an additional 96 h of depuration). Then, 30 mysids were randomly divided into three groups for triplicate experiments (10 mysids per replicate). A total of 10 individuals from each group of 30 mysids were pooled for analysis.

Reactive oxygen species (ROS) target nearby lipids, such as polyunsaturated fatty acids (PUFA). Arachidonic acid, a PUFA, undergoes peroxidation, ultimately forming MDA as a product of lipid peroxidation. MDA is considered a prototype of thiobarbituric acid reactive substances (TBARS). To analyze the intracellular MDA level, the pooled mysids were homogenized using a Teflon homogenizer in a cold buffer containing 20 mM Tris buffer, 100 μM benzamidine, 2 μM aprotinin, 150 mM NaCl, 10 mM β-mercaptoethanol, and 20 μM leupeptin. After centrifugation at 30,000× *g* for 30 min at 4 °C, the collected supernatant was denatured at 75 °C for 15 min. The TBARS content was measured at an excitation wavelength of 535 nm using a Thermo Varioskan Flash spectrophotometer (Thermo Fisher Scientific, Tewksbury, MA, USA). The intracellular MDA levels were quantified with a calibration curve prepared using malondialdehyde bis(dimethyl acetal) (Sigma-Aldrich Co., Burlington, MA, USA) and calculated as nanomoles of MDA per microgram of total protein. Quantification of the total protein was conducted with the Bradford method [34].

The determination of the intracellular GSH level was performed using a Glutathione Assay Kit (Catalog No. CS0260; Sigma-Aldrich Co.). Pooled mysids from each treatment were rinsed with 0.9% NaCl, homogenized in trichloroacetic acid (1:4, *w*/*v*) using a Teflon homogenizer, and then centrifuged at 3000× *g* for 10 min at 4 °C. The supernatant was collected, and the GSH content was measured at 420 nm using the spectrophotometer, following the manufacturer’s protocol. Standard curves were established with GSH equivalents of 0, 150, and 350 μM to measure the total GSH content.

The enzymatic activity levels of CAT and SOD were calculated using specific assay kits (Catalog No. CAT100 and 19160, respectively; Sigma-Aldrich Inc., Chemie, Switzerland). The enzymatic activity levels of GPx and GR were analyzed using the Glutathione Peroxidase Cellular Assay Kit (Sigma-Aldrich Co.) and the Glutathione Reductase Assay Kit (Sigma-Aldrich Inc.), respectively. Their enzymatic activity levels were normalized to the total protein concentration and expressed as units per milligram of total protein.

### 2.5. Measurement of Cholinergic Enzymes

To assess AChE enzymatic activity, acetylthiocholine iodide (ATCh) and 5,5′-dithiobis (2-nitrobenzoic acid) (DTNB) were obtained from Sigma (Sigma-Aldrich Co.). This methodology is based on the biochemical reaction of thiocholine, a product of ATCh hydrolysis, with DTNB, resulting in the formation of 5-mercapto-2-nitrobenzoic acid, which is yellow in color, and its dissociated forms at pH 8. The pooled mysids were homogenized in ice-cold phosphate buffer (0.1 M, pH 8.0) using a Teflon homogenizer at a ratio of 1 part sample to 5 parts buffers (*w*/*v*). The homogenate was then centrifuged at 3000× *g* for 30 min at 4 °C. The supernatant, containing the enzyme, was carefully collected for the AChE assay. For the assay, 100 μL of the supernatant was mixed with 1.3 mL of phosphate buffer (0.1 M, pH 8.0) in a 3 mL cuvette. Additionally, 50 μL of DTNB (0.01 M) and 10 μL of ATCh (0.075 M) were added as substrates. The total AChE enzymatic activity was calculated using the ATCh substrate, along with a control without ATCh and a control without the sample. The measurement, performed using a spectrophotometer, lasted for 5 min at an absorbance of 412 nm and a temperature of 25 °C. The enzymatic activity recorded was normalized to the total protein content in the supernatant.

### 2.6. Statistics

The results were graphically represented using SigmaPlot 10.0 (Systat Software, San Jose, CA, USA). The raw data were processed using SPSS software (version 17.0, SPSS Inc., Chicago, IL, USA) and reported as mean ± standard deviation (S.D.). One-way analysis of variance (ANOVA) was performed, followed by post hoc Tukey and Dennett’s multiple comparison tests to identify statistically significant differences among treatments and control groups. Changes were considered statistically significant if the *p* value was less than 0.05.

## 3. Results

### 3.1. Effect on Survival and Feeding Rates

**Figure 1:**
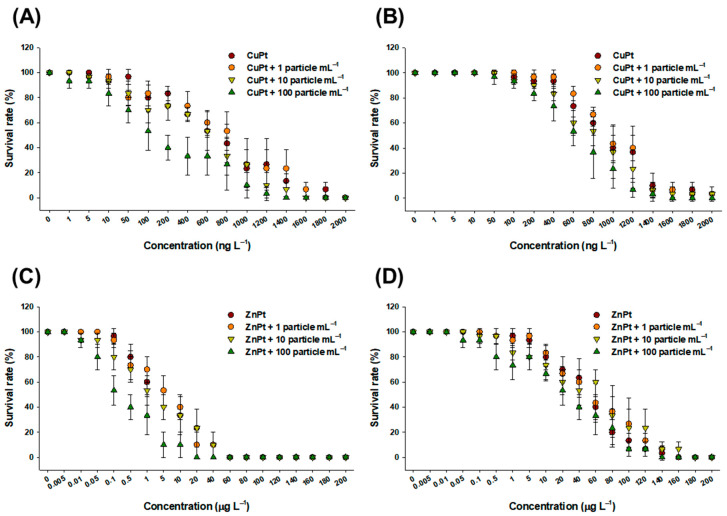
The 96 h survival rates measured in juvenile and adult mysids exposed to CuPT and ZnPT in the absence or presence (1, 10, and 100 particles mL^−1^) of MPs: (**A**) juvenile mysids exposed to CuPT; (**B**) adult mysids exposed to CuPT; (**C**) juvenile mysids exposed to ZuPT; (**D**) adult mysids exposed to ZnPT. Bars indicate the standard deviations of the mean values.

The survival rates from toxicity tests of CuPT and ZnPT, both with and without the three concentrations of MPs, indicate that juvenile mysids are more sensitive to treatment than adults. Overall, the survival rate decreased as the concentration of MPs increased (Figure 1). Exposure to 100 particles mL^−1^ of MPs had the most significant negative impact on survival rate. Higher toxicity was observed in CuPT-exposed mysids compared to ZnPT-exposed mysids.

**Figure 2:**
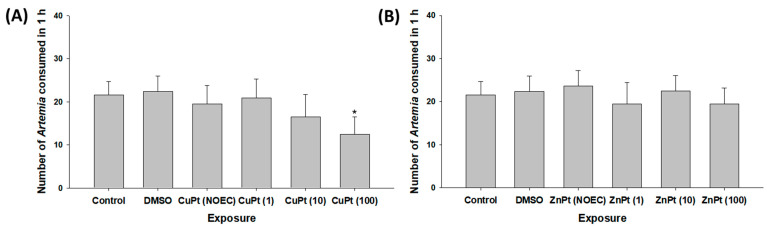
The number of *Artemia* consumed by mysids exposed to (**A**) CuPT and (**B**) ZnPT in the absence or presence (1, 10, and 100 particles mL^−1^) of MPs. The results are presented as mean ± standard deviation (S.D.). An asterisk (*) indicates a significant statistical difference compared to the control value (*p* < 0.05).

Compared to the control, *Artemia* consumption by mysids was significantly lowered after exposure to CuPT with 100 particles mL^−1^ of MPs (*p* < 0.05), whereas other treatments did not significantly affect their feeding rates (*p* > 0.05) (Figure 2A). In the case of ZnPT exposure, no significant fluctuation in feeding activity was observed even when co-exposed with MPs (*p* > 0.05) (Figure 2B).

### 3.2. Oxidative Stress and Response of Antioxidant Parameters

**Figure 3:**
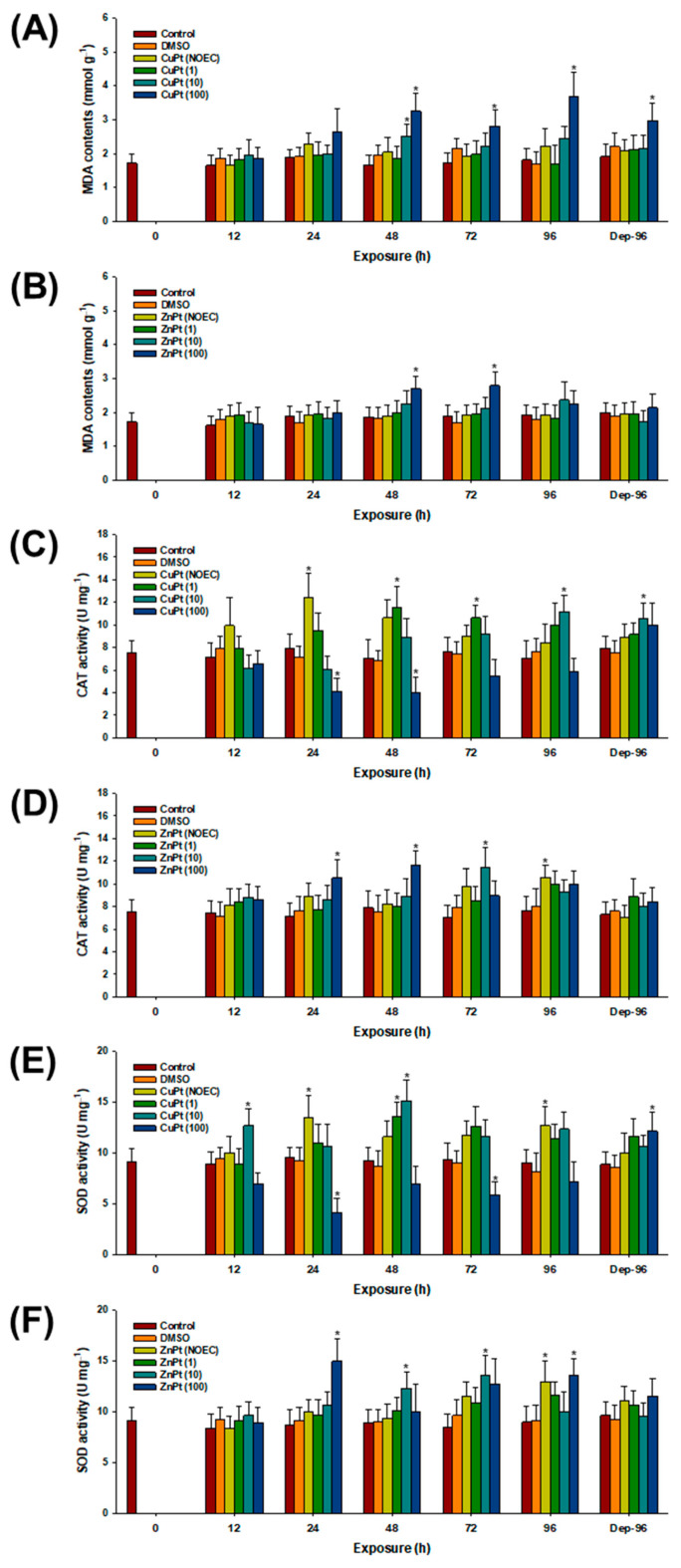
Responses of oxidative stress and antioxidant components represented with intracellular MDA content (**A**,**B**) and enzymatic activity levels of CAT (**C**,**D**) and SOD (**E**,**F**) in mysids exposed to CuPT (**A**,**C**,**E**) and ZnPT (**B**,**D**,**F**). Results are presented as mean ± standard deviation (S.D.). Asterisks (*) indicate significant statistical difference compared to control value (*p* < 0.05).

Significant increases in MDA levels were observed in response to CuPT with 10 particles mL^−1^ of MPs at 48 h (*p* < 0.05) (Figure 3A). The levels were also significantly elevated by CuPT with 100 particles mL^−1^ of MPs at 48, 72, and 96 h, as well as during the 96 h comprising the depuration period (*p* < 0.05). In the case of ZnPT treatment, significantly increased MDA levels were observed with 100 particles mL^−1^ of MPs at 48 and 72 h (*p* < 0.05) (Figure 3B).

Significantly elevated CAT activity was observed in CuPT-exposed mysids at 24 h, even in the absence of MPs (*p* < 0.05) (Figure 3C). The activity was also significantly elevated by CuPT with 10 particles mL^−1^ of MPs at 48, 72, and 96 h, as well as during the 96 h comprising the depuration period (*p* < 0.05), whereas the levels were significantly inhibited by CuPT with 100 particles mL^−1^ of MPs at 24 and 48 h (*p* < 0.05). In the case of ZnPT treatment, significantly elevated CAT activity was observed at 96 h, even in the absence of MPs (*p* < 0.05) (Figure 3D). Significantly increased CAT activity levels were recorded for 10 particles mL^−1^ of MPs at 72 h, as well as by 100 particles mL^−1^ of MPs at 24 and 48 h (*p* < 0.05).

Similarly to the CAT results, significantly elevated SOD activity levels were observed in CuPT-exposed mysids at 24 and 96 h, even in the absence of MPs (*p* < 0.05) (Figure 3E). The activity was also significantly elevated by CuPT with 1 particle mL^−1^ of MPs at 48 h, as well as by 10 particles mL^−1^ of MPs at 12 and 48 h (*p* < 0.05). Significant decreases in SOD activity were detected in response to CuPT with 100 particles mL^−1^ of MPs at 24 and 72 h, whereas increased SOD activity was observed during the 96 h of the depuration period (*p* < 0.05). In the case of ZnPT, the activity was significantly elevated with 1 particle mL^−1^ of MPs at 96 h (*p* < 0.05) (Figure 3F). The levels were also significantly elevated by CuPT with 10 particles mL^−1^ of MPs at 48 and 72 h, as well as by 100 particles mL^−1^ of MPs at 24 and 96 h (*p* < 0.05).

**Figure 4:**
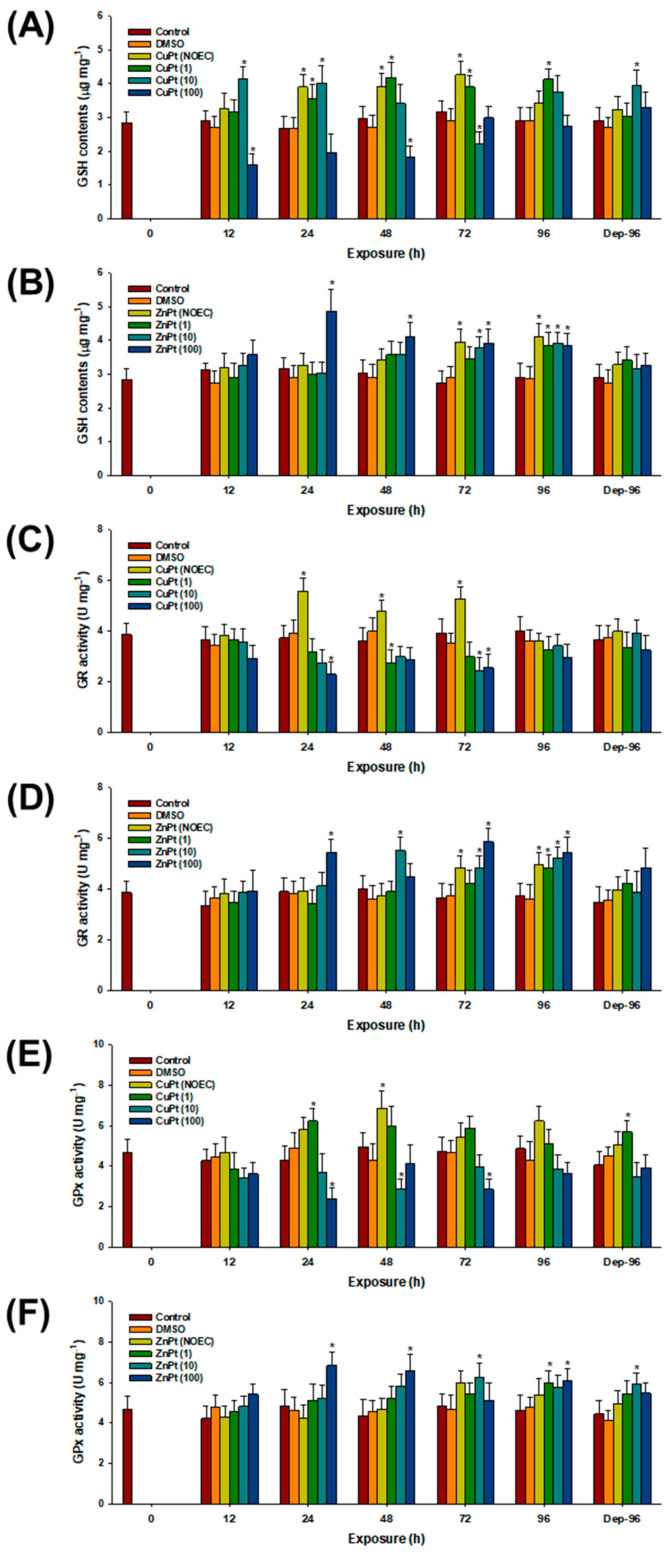
Responses of oxidative stress and antioxidant components represented with intracellular GSH content (**A**,**B**) and enzymatic activity levels of GR (**C**,**D**) and GPx (**E**,**F**) in mysids exposed to CuPT (**A**,**C**,**E**) and ZnPT (**B**,**D**,**F**). Results are presented as mean ± standard deviation (S.D.). Asterisks (*) indicate significant statistical difference compared to the control value (*p* < 0.05).

GSH levels were significantly increased in CuPT-exposed mysids at 24, 48, and 72 h, even in the absence of MPs (*p* < 0.05) (Figure 4A). The contents were also significantly elevated by CuPT with 1 particle mL^−1^ of MPs at 24, 48, 72, and 96 h, as well as by 10 particles mL^−1^ of MPs at 12 and 24 h, in addition to the 96 h of the depuration period (*p* < 0.05). Notably, significant decreases in GSH content were observed with 10 particles mL^−1^ of MPs at 72 h, as well as by 100 particles mL^−1^ of MPs at 12 and 48 h (*p* < 0.05). In the case of ZnPT treatment, significantly increased GSH levels were recorded at 72 and 96 h, even in the absence of MPs (*p* < 0.05) (Figure 4B). The level was also significantly elevated by ZnPT with 1 particle mL^−1^ of MPs at 96 h (*p* < 0.05). Significantly increased GSH contents were observed with 10 particles mL^−1^ of MPs at 72 and 96 h, as well as with 100 particles mL^−1^ of MPs at 24, 48, 72, and 96 h (*p* < 0.05).

Significantly elevated GR activity levels were observed in CuPT-exposed mysids at 24, 48, and 72 h, even in the absence of MPs (*p* < 0.05) (Figure 4C). However, the activity was significantly decreased by CuPT with 1 and 10 particles mL^−1^ of MPs at 48 and 72 h, respectively (*p* < 0.05). In addition, significantly lowered activity levels were observed in response to CuPT with 100 particles mL^−1^ of MPs at 24 and 72 h (*p* < 0.05). In the case of ZnPT treatment, significantly elevated GR activity levels were observed at 72 and 96 h, even in the absence of MPs (*p* < 0.05) (Figure 4D). The activity levels were also elevated by 1 particle mL^−1^ of MPs at 96 h, as well as by 10 particles mL^−1^ of MPs at 48, 72, and 96 h (*p* < 0.05). In addition, significantly elevated GR activity levels were detected by 100 particles mL^−1^ of MPs at 24, 72, and 96 h (*p* < 0.05).

GPx activity was significantly elevated by CuPT at 48 h, even in the absence of MPs (*p* < 0.05) (Figure 4E). The activity was also significantly elevated by CuPT with 1 particle mL^−1^ of MPs at 48 h, as well as during the 96 h depuration period (*p* < 0.05). However, significantly decreased activity levels were observed with 10 particles mL^−1^ of MPs at 48 h, as well as by 100 particles mL^−1^ of MPs at 24 and 72 h (*p* < 0.05). In the case of ZnPT, the activity was significantly elevated with 1 particle mL^−1^ of MPs at 96 h (*p* < 0.05) (Figure 4F). The levels were also significantly elevated by 10 particles mL^−1^ of MPs at 72 h, as well as during the 96 h depuration period, and by 100 particles mL^−1^ of MPs at 24, 48, and 96 h (*p* < 0.05).

### 3.3. Effect on AChE Activity

**Figure 5:**
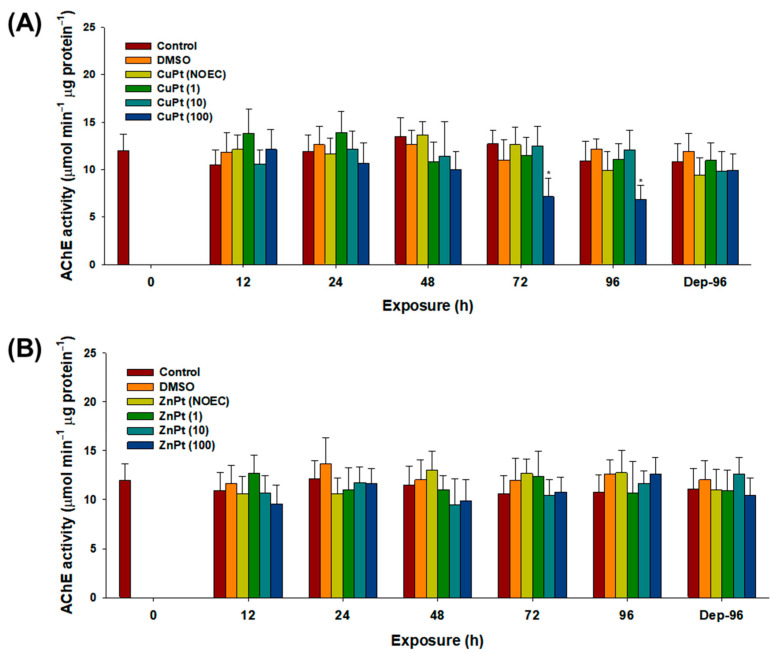
Cholinergic effect, measured as acetylcholinesterase activity, in mysids exposed to CuPT and ZnPT. (**A**) CuPT; (**B**) ZnPT. Results are presented as mean ± standard deviation (S.D.). Asterisks (*) indicate significant statistical difference compared to control value (*p* < 0.05).

The enzymatic activity levels of AChE were significantly decreased by CuPT with 100 particles mL^−1^ of MPs at 72 and 96 h (*p* < 0.05) (Figure 5A). However, no significant modulation was observed with ZnPT in the absence or presence of MPs (*p* > 0.05) (Figure 5B).

## 4. Discussion

While the toxic impacts of CuPT, ZnPT, and MPs on marine animals have been studied previously, the combined effects of these pollutants on aquatic animals, particularly at sub-lethal levels, are poorly understood. To our knowledge, our results comprise the first report on the combined toxic effects of representative antifoulants and MPs on the acute response of small zooplankton. Co-exposure with MPs clearly induced concentration-dependent additional or synergistic effects on the acute toxicity of CuPT and ZnPT in both stages of *N. awatchensis*. Although not drastically different, there was a trend of increased mortality in *N. awatchensis* with elevated concentrations of MPs. Due to their surface characteristics and hydrophobicity, increasing evidence has suggested that MPs can serve as vectors for organic and inorganic pollutants [20,21,22,35,36]. The ingestion of polystyrene MPs ranging from 1 to 10 µm has been confirmed in mysid species [33,37]. Although most ingested polystyrene MPs are egested through feces, clogged and embedded MPs have been consistently observed in the internal digestive systems of *N. awatchensis* [33]. Therefore, higher ingestion of MPs can induce the bioconcentration of CuPT and ZnPT in the internal organs of mysids, intensifying their toxicity. The internal organs are not the sole target of MPs, as they can also attach to the external bodies of mysids, such as the setae and gaps between plates. Attachment to the external body has been confirmed in the mysid *Mesopodopsis orientalis* [38]. In *N. awatchensis*, the highest concentration of MPs, 100 particles mL^−1^, had no significant effect on survival [33]. Therefore, we assume that MPs have additive or synergistic effects on the toxicity of CuPT and ZnPT through temporal bioconcentration and the transfer of the antifoulants into the body, beyond the sole effects of the plastic particles.

Food consumption is a crucial, fundamental, and essential behavior for the fitness and general physiology of organisms. Feeding in mysids relies on the many movable parts of their complex mouth structures, but it has been confirmed that MPs ranging from 1 to 10 µm can be easily ingested into their digestive systems without significant internal clogging [33,37]. The results of field investigations have revealed that diverse MP types even ranging from 58 to 4669 μm have been extracted from the internal bodies of mysids [38]. Therefore, we assume that the sizes and concentrations of the MPs used in this study would not be critical for the inhibition of feeding activity. The lack of significant effects of ZnPT can be explained by the different sensitivities of *N. awatchensis* to both antifoulants. The 96 h LC50 values for CuPT were 441 ng L^−1^ for juveniles and 785 ng L^−1^ for adults, whereas the values for ZnPT were 3.28 μg L^−1^ for juveniles and 28.63 μg L^−1^ for adults [15]. Reduced feeding activity due to xenobiotics has been associated with decreased swimming behavior, lowered detoxification capacity, growth retardation, and increased mortality [15,33,39,40]. Since feeding is the intake of nutrients, a decline in feeding activity can lead to an insufficient energy supply for movement, development, growth, fertility, and reproduction, and ultimately result in mortality in mysids.

Aquatic organisms living in aerobic environments have evolved various antioxidant mechanisms to counteract the continuous production of intracellular ROS and maintain cellular homeostasis [29,30,31]. Overall, the fluctuation in antioxidants observed in this study suggests an increase in oxidative stress, which can be triggered by either excessive production of ROS or inadequate removal of free radicals in response to the additive or synergistic effects of antifoulants and MPs. The excessive ROS can then propagate throughout the cell and damage major intracellular constituents, such as lipids. MDA, a final product of lipid peroxidation, is commonly measured to assess lipid modification and serves as an indicator of oxidative stress. MDA is essentially formed through the degradation of fatty acids by excessive ROS, leading to significant damage (e.g., cell membrane injury) [30]. The significant elevation in MDA detected in *N. awatchensis* suggests the induction of oxidative stress due to exposure to antifoulants and MPs. An impaired membrane makes the organism inherently more vulnerable to cellular toxicity by compromising essential biochemical processes such as signal transduction and ion exchange [41,42]. The elevated MDA content measured during the depuration period of *N. awatchensis* exposed to CuPT and MPs suggests the higher toxicity of CuPT compared to ZnPT.

SOD is an antioxidant enzyme that converts the superoxide anion (O_2_^−^) into less reactive species, specifically H_2_O_2_ and O_2_ [43]. CAT plays a role in detoxifying H_2_O_2_ further by breaking it down into H_2_O and O_2_ and, thus, it eliminates further adverse impact [30]. Given their pivotal role in the first line of antioxidant response, the notable increases in enzyme activity are likely a response to the heightened intracellular ROS levels. This supports the notion that the simultaneous exposure to antifoulants and MPs triggers oxidative stress in *N. awatchensis*. Notably, significantly reduced enzymatic activity levels of CAT and SOD were observed in *N. awatchensis* exposed to CuPT in the presence of MPs. Considering the higher toxicity of CuPT compared to ZnPT in this species, the measured reductions in the enzymatic activity levels of CAT and SOD suggest a significant induction of oxidative stress by CuPT. This oxidative stress could impair the enzymatic functions, ultimately rendering *N. awatchensis* more susceptible to oxidative damage.

The significant increases in GSH levels and the increased GPx and GR activity levels appear to be protective responses triggered by elevated ROS levels. GSH serves as a non-enzymatic antioxidant by supporting the enzymatic antioxidant system as a cofactor [30]. GPx facilitates the breakdown of H_2_O_2_ into H_2_O and O_2_ using GSH as a substrate [31]. GR catalyzes the reduction of glutathione disulfide (GSSG) to GSH, utilizing NADPH as an electron donor, thereby maintaining cellular redox balance [43]. This cycle ensures that GSH, once oxidized, is regenerated to continue neutralizing H_2_O_2_ [44]. Therefore, their increases suggest strong oxidative stress induced by individual antifoulants or their co-exposure with MPs, as well as the active involvement of the GSH-mediated antioxidant response to diminish oxidative stress in *N. awatchensis*. However, combined exposure to CuPT and MPs significantly reduced the GSH-mediated defense system. Under oxidative stress, GSH is rapidly utilized to scavenge peroxides via GPx, generating GSSG as a byproduct. The lowered GSH levels should be replenished by GR, reducing GSSG back to GSH. GSH can be depleted by the prolonged use and/or reduced enzymatic activity levels of GPx and GR. Thus, GSH depletion is an early indicator of various cell death mechanisms [45]. Given that CuPT is more toxic than ZnPT, GSH content can become depleted, contributing to the different sensitivities of *N. awatschensis*. Notably, all components of the GSH-mediated defense system were reduced in the groups co-exposed to MPs, indicating that MPs have an additive or synergistic effect on CuPT toxicity.

The basic function of AChE terminates neurotransmission by hydrolyzing ACh at the postsynaptic junctions. If the enzyme fails to participate in this biochemical reaction, ACh accumulates, leading to the overstimulation of nicotinic and muscarinic receptors [46]. This overstimulation results in muscle fasciculations, neuromuscular blockades, and bradycardia [47]. In *N. awatschensis*, the significant decrease in AChE activity when exposed to CuPT combined with MPs indicates a potential disruption of cholinergic neurotransmission. It was also revealed that MPs have clear additive or synergistic effects on CuPT toxicity, as evidenced by the results of the antioxidant defense system. In aquatic invertebrates, such additive or synergistic effects of MPs on xenobiotic toxicities have been consistently reported [48,49,50,51,52,53]. The reduction in AChE activity is likely associated with the observed decrease in the feeding rate of *N. awatschensis*. Reduced muscle contraction resulting from AChE inhibition could account for the decline in motor function, manifesting as reduced feeding activity.

Generally, co-exposure to MPs and organic pollutants is known to be more toxic than exposure to dissolved pollutants alone [18,19,20], as confirmed by our research. Although virgin plastics have relatively low reactivity compared to weathered plastics, the combined harmful effects of MPs with CuPT and ZnPT have been clearly observed in marine mysids. This suggests that MPs can act as carriers for biofoulants. The sorption of biofoulants onto the surfaces of MPs and their desorption kinetics are likely crucial factors for additive and/or synergistic effects, while leaching potential is less significant in this study due to the use of pristine MPs over a short time period. Additionally, it is important to note that our study utilized sub-lethal concentrations of biofoulants and MPs, as many studies have demonstrated additive and/or synergistic effects of MPs at concentrations much higher than those typically found in natural conditions. Thus, our findings contribute to the understanding of the joint toxicity of MPs and organic pollutants in marine environments. However, further investigation is needed to explore the interactions between MPs and various organic pollutants and their combined effects on marine organisms.

Although it has been demonstrated that MPs can enhance the toxicity of antifoulants through additive and/or synergistic effects, several limitations should be addressed in future research, as this study utilized commercial polystyrene MPs. Commercial pristine MPs do not accurately represent environmental conditions due to their lack of environmental weathering and their uniform chemical and physical properties. The consistent sizes and shapes of commercial MPs also do not reflect the variability found in natural environments. These factors can affect how microplastics are ingested, distributed, and metabolized by organisms, differing from real-world scenarios. To more accurately assess the impact of MPs, future studies should use environmentally relevant MPs in toxicity tests.

## 5. Conclusions

In summary, this study highlights the physiological and biochemical changes in *N. awatschensis* due to combined exposure to two representative antifoulants along with MPs, resulting in significant oxidative stress and neurotoxicity. The observed fluctuations in their antioxidant defense systems, along with their increased MDA levels, underscore the oxidative stress imposed on *N. awatschensis* by the additive or synergistic effects of MPs on the antifoulants. Exposure to MPs revealed the differing sensitivity to antifoulants in this species, with CuPT mixed with MPs showing a higher toxic potential compared to ZnPT mixed with MPs. The combined harmful effects of MPs with CuPT and ZnPT on mysids could have significant environmental implications, potentially impacting the mysid population and food web. These findings emphasize the importance of considering the combined effects of multiple pollutants with MPs, as their interactions can have more severe adverse impacts on aquatic organisms than when assessed individually. Further research is needed on the leaching potentials of MPs, as well as their bioaccumulation and bioavailability in this species.

## Data Availability

Data can be made available upon reasonable request.
